# An atlas of genetic correlations between psychiatric disorders and human blood plasma proteome

**DOI:** 10.1192/j.eurpsy.2019.6

**Published:** 2020-02-20

**Authors:** Shiqiang Cheng, Fanglin Guan, Mei Ma, Lu Zhang, Bolun Cheng, Xin Qi, Chujun Liang, Ping Li, Om Prakash Kafle, Yan Wen, Feng Zhang

**Affiliations:** 1 Key Laboratory of Trace Elements and Endemic Diseases of National Health and Family Planning Commission, School of Public Health, Health Science Center, Xi’an Jiaotong University, Xi’an, China; Collaborative Innovation Center of Endemic Diseases and Health Promotion in Silk Road Region, Xi’an Jiaotong University, Xi’an 710061, China; 2 School of Medicine & Forensics, Health Science Center, Xi’an Jiaotong University, Xi’an, China

**Keywords:** Genetic correlation, genome-wide association study, linkage disequilibrium score regression, plasma proteins, psychiatric disorders

## Abstract

**Background.:**

Psychiatric disorders are a group of complex psychological syndromes with high prevalence. Recent studies observed associations between altered plasma proteins and psychiatric disorders. This study aims to systematically explore the potential genetic relationships between five major psychiatric disorders and more than 3,000 plasma proteins.

**Methods.:**

The genome-wide association study (GWAS) datasets of attention deficiency/hyperactive disorder (ADHD), autism spectrum disorder (ASD), bipolar disorder (BD), schizophrenia (SCZ) and major depressive disorder (MDD) were driven from the Psychiatric GWAS Consortium. The GWAS datasets of 3,283 human plasma proteins were derived from recently published study, including 3,301 study subjects. Linkage disequilibrium score (LDSC) regression analysis were conducted to evaluate the genetic correlations between psychiatric disorders and each of the 3,283 plasma proteins.

**Results.:**

LDSC observed several genetic correlations between plasma proteins and psychiatric disorders, such as ADHD and lysosomal Pro-X carboxypeptidase (*p* value = 0.015), ASD and extracellular superoxide dismutase (Cu-Zn; *p* value = 0.023), BD and alpha-N-acetylgalactosaminide alpha-2,6-sialyltransferase 6 (*p* value = 0.007), MDD and trefoil factor 1 (*p* value = 0.011), and SCZ and insulin-like growth factor-binding protein 6 (*p* value = 0.011). Additionally, we detected four common plasma proteins showing correlation evidence with both BD and SCZ, such as tumor necrosis factor receptor superfamily member 1B (*p* value = 0.012 for BD, *p* value = 0.011 for SCZ).

**Conclusions.:**

This study provided an atlas of genetic correlations between psychiatric disorders and plasma proteome, providing novel clues for pathogenetic and biomarkers, therapeutic studies of psychiatric disorders.

## Introduction

Psychiatric disorders are a group of complex psychological symptoms, mainly characterized by clinically significant deficits in an individuals’ cognition, emotion regulation, and behavior [1]. The common psychiatric disorders include the attention deficiency/hyperactive disorder (ADHD), autism spectrum disorder (ASD), bipolar disorder (BD), schizophrenia (SCZ), and major depressive disorder (MDD). Psychiatric disorders have been found commonly, with over a third of people in most countries reporting sufficient criteria to be diagnosed at some points of their lives [2]. Epidemiological research has shown that about 3–18% of children suffered psychiatric disorders causing significant functional impairment [3].

Psychiatric disorders are considered multifactorial and driven by a combination of biological, psychological, and environmental factors [4]. Multiple epidemiological [5,6] and molecular biological [7,8] studies have observed shared risk components among various psychiatric disorders. Similar environmental risk factors like physical abuse and neglect have also been found to underlie a range of psychiatric disorders, for instance SCZ and depression [9]. Recently, there is a growing body of researches focus on the genetic mechanism of psychiatric disorders. The implication of genetic factors in the pathogenesis of psychiatric disorders has been well documented. For instance, the estimated heritability achieved 80% for SCZ [10] and >90% for classic autism [11]. Multiple large-scale genetic studies of psychiatric disorders have been conducted and identified multiple susceptibility genes for psychiatric disorders [12]. However, the etiology and molecular mechanism of psychiatric disorders remains elusive now.

Plasma proteins (also named blood proteins) are a group of proteins in blood plasma. More than 3,600 plasma proteins have been discovered, functionally implicated in signaling, transport, repair, and defense against infection [13]. Altered plasma proteins have been found to be related to multiple human complex diseases including psychiatric disorders [14,15]. As important intermediate phenotypes, plasma proteins are useful for early disease diagnosis, understanding human physiology, developing health biomarkers, and targeting to therapy [16,17]. For instance, Hye et al. found that complement factor H and α-2-macroglobulin were specific markers of Alzheimer’s disease [18]. More recently, a study suggested that apolipoprotein A-1 could act as a serum marker for the response to lithium treatment in BD [19]. However, few efforts were paid to systematically explore the relationships between psychiatric disorders and plasma proteome.

It is well known that gene expression is under genetic control [20]. Extensive efforts have been paid to explore the genetic mechanism of gene expression regulation and identified a lot expression quantitative trait loci (eQTLs) [21]. Recently, Foss et al. performed a large-scale genome-wide association study of more than 3,000 plasma proteins [22]. They identified a group of significant protein quantitative trait loci (pQTLs) associated with plasma proteins levels [22]. They also observed that the effects of eQTLs on transcript differed from that on protein levels, which emphasizes the importance of pQTLs studies [22].

Recent studies demonstrated the generality of genetic correlations among complex human phenotypes. Linkage disequilibrium score (LDSC) regression is an efficient method and widely used for evaluating the genetic relationships among different human phenotypes [23]. Utilizing genome-wide association study (GWAS) summary data, LDSC provides an easy and reliable way to simultaneously screen thousands of traits and find out the real genetic correlations among them [24]. Utilizing LDSC, Bulik-Sullivan et al. evaluated 276 genetic correlations among 24 traits, and observed significant genetic correlations between anorexia nervosa and SCZ, anorexia and obesity, and educational attainment and several diseases [25]. Duncan et al. suggested that LDSC was an appropriate polygenic methods to estimate the overlapping genetic factors between post‐traumatic stress disorder (PTSD) and SCZ as well as bipolar and MDD [26].

In this study, utilizing the latest GWAS data of blood proteins and five common psychiatric disorders from the Psychiatric Genomics Consortium (PGC), LDSC was used to systematically evaluate the genetic relationships between five common psychiatric disorders and human plasma proteome.

## Materials and Methods

### GWAS datasets of five psychiatric disorders

The latest GWAS summary data of ADHD (19,099 cases and 34,194 controls), ASD (7,387 cases and 8,567 controls), BD (20,129 cases and 21,524 controls), SCZ (33,426 cases and 32,541 controls), and MDD (135,458 cases and 344,901 controls) were downloaded from the Psychiatric GWAS Consortium (PGC) website (https://www.med.unc.edu/pgc/results-and-downloads) as discovery samples [27–30]. Briefly, all study subjects were European whites and diagnosed using research standard diagnoses and expert clinical consensus diagnosis. Genotyping was performed using commercial platform such as Illumina 610K and Affymetrix SNP 6.0 chips. Imputation was conducted using IMPUTE2 against public reference panels such as the 1,000 Genomes Project Phase 2 and Phase 3. Association analysis was conducted using logistic regression model. Detailed description of sample characteristics, experimental design, and statistical analysis can be found in the published studies [27-30].

### Cross-disorder GWAS replication data of psychiatric disorders

The latest cross-disorder GWAS of six common psychiatric disorders was derived from the Lundbeck Foundation Initiative for Integrative Psychiatric Research (iPSYCH) [31]. Briefly, Schork et al. conducted a cross-disorder GWAS of six common psychiatric disorders, including ADHD, anorexia, ASD, affective disorder, BP, and SCZ [31]. The total sample size included 65,534 individuals with 46,008 cases and 19,526 controls [31]. SNP genotying was performed by Inifinium PsychChip v1.0 array. Imputation was conducted using Impute2 with the 1,000 genomes project phase 3 reference. GWAS summary statistics were computed using logistic regression of the plink software. Age, gender, and 10 principle components of population structure were included as covariates. Detailed description of sample characteristics, genotyping, imputation, experimental design, and statistical analysis can be found in the published studies [31].

### pQTL data of human plasma proteome

The GWAS summary data of human plasma proteome were derived from a recently published study [13]. Briefly, Sun et al. quantify 3,622 plasma proteins in 3,301 healthy participants from the INTERVAL [32] study by using an expanded version of an aptamer-based multiplex protein assay (SOMAscan) [13]. The genotyping protocol and quality control for the INTERVAL samples have been described previously in detail [33]. Briefly, genotyping was performed on the Affymetrix Axiom UK Biobank genotyping array. Imputation was performed via the Sanger Imputation Server by using a combined 1,000 Genomes Phase 3-UK10K reference panel. Simple linear regression using an additive genetic model was used to test genetic associations. After quality control, the GWAS summary data of 3,283 plasma proteins were used in following genetic correlation analysis. Detailed description of sample characteristics, experimental design, quality control, and statistical analysis can be found in the published studies [13].

### Genetic correlation scanning

Following the approach recommended by the developers [23,34] and previous study [25], LDSC software (v1.0.0; https://github.com/bulik/ldsc) were applied to the GWAS summary data for evaluating the genetic correlations between each of the five psychiatric disorders and each of the 3,283 plasma proteins at first. Using the same method, the significant genetic correlations were further validated using the cross-disorder GWAS replication data. The basic principle of the LDSC approach is to estimate directly from GWAS summary data using the deviation of the observed *χ* ^2^ test statistic for a SNP from its expected value under the null hypothesis of no association [35]. An SNP tagging more of its neighbors—and, thus, having a higher LD score—is more likely to tag one or more causal sites affecting the phenotype [35]. If genetic correlations are statistically and quantitatively significant, then we can determine that total phenotypic correlations cannot be attributed to fully environmental confounders [24]. In addition, Anney et al. have demonstrated that LD score regression can distinguish genuine polygenicity from the bias caused by population stratification and cryptic relatedness [28]. The European LD scores, calculated from the 1,000 Genomes by the developers, were used in this study [28].

## Results

LDSC regression observed several genetic correlation signals between plasma proteins and psychiatric disorders with LDSC *p* values <0.05. For ADHD, genetic correlation signals were observed for lysosomal Pro-X carboxypeptidase (coefficient = 0.243, *p* value = 0.015), and alpha-2-antiplasmin (coefficient = 0.274, *p* value = 0.032).

For ASD, genetic correlations were observed for extracellular superoxide dismutase (Cu-Zn; coefficient = 0.530, *p* value = 0.023), hepatitis A virus cellular receptor 1 (coefficient = 0.405, *p* value = 0.031), chromogranin-A (coefficient = 0.409, *p* value = 0.034), pro-opiomelanocortin (POMC; coefficient = 0.523, *p* value = 0.041), cysteine-rich hydrophobic domain-containing protein 2 (coefficient = 0.263, *p* value = 0.043), and trypsin-1 (coefficient = 0.397, *p* value = 0.047).

Nine plasma proteins were detected for BD such as alpha-N-acetylgalactosaminide alpha-2,6-sialyltransferase 6 (coefficient = 0.419, *p* value = 0.007), tumor necrosis factor receptor superfamily member 1B (coefficient = −0.383, *p* value = 0.012), guanine nucleotide exchange factor VAV3 (coefficient = −0.270, *p* value = 0.018), insulin-like growth factor-binding protein 6 (coefficient = −0.377, *p* value = 0.022), and rho guanine nucleotide exchange factor 10 (coefficient = −0.304, *p* value = 0.022).

For MDD, 12 blood plasma proteins were detected such as trefoil factor 1 (coefficient = −0.287, *p* value = 0.011), bone morphogenetic protein 7 (coefficient = 0.392, *p* value = 0.012), peregrin (coefficient = 0.361, *p* value = 0.013), beta-defensin 118 (coefficient = 0.328, *p* value = 0.014), and Neurensin-1 (coefficient = −0.359, *p* value = 0.026).

For SCZ, 15 blood plasma proteins were detected such as insulin-like growth factor-binding protein 6 (coefficient = −0.396, *p* value = 0.011), cathepsin Z (coefficient = −0.349, *p* value = 0.012), sphingosine kinase 2 (coefficient = 0.212, *p* value = 0.018), tropomyosin alpha-1 chain (coefficient = 0.264, *p* value = 0.021), CMP-N-acetylneuraminate-beta-galactosamide-alpha-2,3-sialyltransferase 1 (coefficient = 0.269, *p* value = 0.028), and protein nephroblastoma overexpressed (NOV) homolog (coefficient = 0.196, *p* value = 0.028; Table 1).

**Table 1.** Genetic correlations analysis results between psychiatric disorders and blood plasma protein (*p* value <0.05)

Psychiatric disorders	Blood plasma protein	Coefficients	*p* values
ADHD	Lysosomal Pro-X carboxypeptidase	0.243	0.015
	Alpha-2-antiplasmin	0.274	0.032
ASD	Extracellular superoxide dismutase (Cu-Zn)	0.530	0.023
	Hepatitis A virus cellular receptor 1	0.405	0.031
	Chromogranin-A	0.409	0.034
	Pro-opiomelanocortin	0.523	0.041
	Cysteine-rich hydrophobic domain-containing protein 2	0.263	0.043
	Trypsin-1	0.370	0.047
BD	Alpha-N-acetylgalactosaminide alpha-2,6-sialyltransferase 6	0.419	0.007
	Tumor necrosis factor receptor superfamily member 1B	−0.383	0.012
	Guanine nucleotide exchange factor VAV3	−0.270	0.018
	Insulin-like growth factor-binding protein 6	−0.377	0.022
	Rho guanine nucleotide exchange factor 10	−0.304	0.022
	Sodium-coupled monocarboxylate transporter 1.sumstats	−0.210	0.027
	Normal mucosa of esophagus-specific gene 1 protein	−0.215	0.030
	Muscle, skeletal receptor tyrosine-protein kinase	−0.200	0.033
MDD	Trefoil factor 1	−0.287	0.011
	Bone morphogenetic protein 7	0.392	0.012
	Peregrin	0.361	0.013
	Beta-defensin 118	0.328	0.014
	Neurensin-1	−0.359	0.026
	Multimerin-2	0.322	0.026
	Matrix metalloproteinase-17	0.358	0.026
	Intercellular adhesion molecule 3	0.241	0.036
	Tumor necrosis factor receptor superfamily member 8	0.297	0.040
	Neurotensin/neuromedin N	0.313	0.041
	Cyclin-dependent kinase 2:Cyclin-A2 complex	0.443	0.045
	Glyceraldehyde-3-phosphate dehydrogenase, testis-specific	−0.336	0.048
	Trefoil factor 1	−0.287	0.011
SCZ	Insulin-like growth factor-binding protein 6	−0.396	0.011
	Cathepsin Z	−0.349	0.012
	Sphingosine kinase 2	0.212	0.018
	Tropomyosin alpha-1 chain	0.264	0.021
	CMP-N-acetylneuraminate-beta-galactosamide-alpha-2,3-sialyltransferase 1	0.269	0.028
	Protein NOV homolog	0.196	0.028
	Normal mucosa of esophagus-specific gene 1 protein	−0.167	0.030
	Alpha-L-iduronidase	−0.260	0.032
	Potassium voltage-gated channel subfamily E member 2	0.182	0.039
	Cell surface glycoprotein CD200 receptor 2	−0.233	0.041
	Profilin-2	−0.329	0.042
	Ubiquitin-conjugating enzyme E2 T	0.264	0.043
	Rho guanine nucleotide exchange factor 10	−0.229	0.044
	Tumor necrosis factor receptor superfamily member 1B	−0.292	0.045
	Disintegrin and metalloproteinase domain-containing protein 29	0.272	0.048

Abbreviations: ADHD, attention deficiency/hyperactive disorder; ASD, autism spectrum disorder; BD, bipolar disorder; MDD, major depressive disorder; SCZ, schizophrenia.

After comparing the LDSC results of the five psychiatric disorders, we also detected four common plasma proteins shared by BD and SCZ, including tumor necrosis factor receptor superfamily member 1B (*p* value = 0.012 for BD, *p* value = 0.011 for SCZ), insulin-like growth factor-binding protein 6 (*p* value = 0.022 for BD, *p* value = 0.030 for SCZ), rho guanine nucleotide exchange factor 10 (*p* value = 0.022 for BD, *p* value = 0.044 for SCZ), and normal mucosa of esophagus-specific gene 1 protein (*p* value = 0.030 for BD, *p* value = 0.045 for SCZ; Table 2).

**Table 2.** Common genetic correlations between psychiatric disorders and plasma protein (*p* < 0.05).

Blood plasma protein	*p* values	
	BD	SCZ
Tumor necrosis factor receptor superfamily member 1B	0.012	0.011
Insulin-like growth factor-binding protein 6	0.022	0.030
Rho guanine nucleotide exchange factor 10	0.022	0.044
Normal mucosa of esophagus-specific gene 1 protein	0.030	0.045

Abbreviations: BD, bipolar disorder; SCZ, schizophrenia.

The significant genetic correlations detected in the discovery GWAS datasets of five psychiatric disorders were further validated in the cross-disorder replication GWAS data. Two proteins identified in the discovery GWAS were further replicated in the cross-disorder replication GWAS data including multimerin-2 (coefficient = 0.471, *p* value = 0.032) and tumor necrosis factor receptor superfamily member 8 (coefficient = 0.388, *p* value = 0.033).

## Discussion

To provide an atlas of genetic correlations between psychiatric disorders and plasma proteins, we conducted a large-scale genetic correlations between five common psychiatric disorders and 3,283 plasma proteins. We observed modest genetic correlations and identified several plasma proteins showing genetic correlation evidence with the five psychiatric disorders. Our study results provide novel clues for the pathogenetic and biomarkers studies of common psychiatric disorders.

We found that POMC was correlated with autism, which was consistent with previous study [36]. POMC is a precursor polypeptide with 241 amino acid residues, and cleave to give rise to multiple peptide hormones. Previous studies of adult individuals exhibiting self-injurious behavior suggested that the pro-opiomelanocortin system, especially the endogenous opioid system, was dysregulated in the subgroups of autistic patients [37,38]. Cazzullo et al. have suggested that the concentration of plasma POMC fragments, especially opioid fragments, contributed to the symptoms of autism as well as the response to treatment [39]. A mutation in the opioid region of the POMC gene in an autistic individual indicated that a subgroup of patients will be identified who share a POMC genetic defect [40,41].

Bone morphogenetic protein 7 (BMP7), a member of the transforming growth factor-β superfamily, is another notable finding of this study. BMP7 plays a critical role in the development of noradrenergic neurons. It has neurotrophic and neuroprotective effects on mature catecholaminergic neurons [42,43]. Real-time polymerase chain reaction (PCR) of locus coeruleus tissue from 12 matched pairs of MDD subjects and psychiatrically normal control subjects revealed low levels of BMP7 gene expression in MDD [44]. Laser capture microdissection of noradrenergic neurons, astrocytes, and oligodendrocytes from the locus coeruleus revealed that the MDD-associated reduction in BMP7 gene expression was limited to astrocytes [44]. This suggests that reduced astrocyte support for pontine locus coeruleus neurons may contribute to pathology of brain noradrenergic neurons in MDD [44]. Rats exposed to chronic social defeat exhibited a similar reduction in BMP7 gene expression in the locus coeruleus [44].

The defects responsible for impaired sensorimotor gating in mice, a hallmark of SCZ, might include myelination dysregulation, which has been observed in some cases of human SCZ [45,46]. Notably, sphingosine 1 phosphate (S1P) receptor expression in oligodendrocytes involves in the process of myelination in the rodent central nervous system and might contribute to glial differentiation, maturation, and myelination during development [47]. Contos et al. suggested that constitutive knockout of S1P receptor 1 causes a behavioral phenotype reminiscent of SCZ [48]. The concentration of S1P is regulated by the activities of two kinases—sphingosine kinase 1 and 2, a number of broad specificity lipid phosphate phosphatases which have a selectivity toward S1P [49]. In this study, we found that sphingosine kinase 2 was correlated with SCZ, which is consistent with previous conclusions.

Additionally, we observed genetic correlation evidence between guanine nucleotide exchange factor 3 VAV3 (VAV3) and BD. A study including 199 participants from the Mayo Clinic Bipolar Disorder Biobank suggested that several SNPs of VAV3 gene was associated with the response to antiepileptic drugs-mood stabilizers in BD patients [50]. Previous molecular biological studies have found multiple shared risk components between BD and SCZ [5,7]. It is interesting that we found common proteins shown significant genetic correlations between BD and SCZ in this study, for instance tumor necrosis factor receptor superfamily member 1B (TNFRSF1B). TNFRSF1B, also known as tumor necrosis factor receptor 2 (TNFR2), is a membrane receptor that binds tumor necrosis factor-alpha. TNFRSF1B is expressed in glia and neurons [51]. It has been reported that TNFRSF1B mediated trophic or protective role in neuronal survival [52]. TNFRSF1B knockout studies in mice suggested a role of TNFRSF1B in protecting neurons from apoptosis by stimulating antioxidative pathways [53]. Till et al. suggested that the polymorphism of TNFRSF1B gene resulted in a lower capability to induce NF-kB activation, leading to an enhancement of TNFR1-induced apoptosis [54]. SCZ patients with 676G allele of TNFRSF1B have a decreased neuron survival, dendritic branching, and capacity of remyelination [55]. Compared with healthy control subjects, SCZ and BD patients have higher plasma soluble TNFRSF1B levels [56]. This could be interpreted as the increasing in soluble TNFRSF1B levels to reduce apoptosis and modulate TNF activity in the euthymic period in BD [57]. Based on the previous and our study results, it is reasonable to infer that the observed negative genetic correlations between TNFRSF1B and BD/SCZ may partially be explained by the protective effects of TNFRSF1B on neurons. Further studies are needed to confirm the role of TNFRSF1B in the pathological mechanisms of BD and SCZ.

To the best of our knowledge, this is the first large-scale genetic correlation analysis of plasma proteome and psychiatric disorders. Because of using GWAS genetic data, our study results should be less susceptible to environmental confounding factors. Notably, two limitations of our approach should also be noted. First, it should be noted that the objective of this study is to evaluate the genetic correlations between plasma proteome and psychiatric disorders, and to scan novel candidate plasma proteins related to psychiatric disorders. Further functional studies are needed to confirm our findings and clarify the potential biological mechanisms of observed associations between plasma proteins and psychiatric disorders in this study. Second, the GWAS summary data of this study are all from European ancestry. Therefore, it should be careful to apply our study results to other ethnic groups.

## Conclusions

In summary, by utilizing LDSC approach, we conducted a large-scale analysis to investigate the genetic correlations between blood plasma proteome and psychiatric disorders. Our study identified a set of candidate plasma proteins showing association signals with psychiatric disorders. We hope that our findings will provide novel insights into the future pathogenetic studies of psychiatric disorders and serve as a fundamental resource for understanding the genetic mechanisms of the effects of plasma proteome on psychiatric disorders.
